# Foliar Application of Nanoselenium Enhances Drought Tolerance in *Brassica oleracea* var. *italica* Through Antioxidant Reinforcement and Pigment Stabilization

**DOI:** 10.3390/life16010070

**Published:** 2026-01-02

**Authors:** Simona Ioana Vicas, Cristina Adriana Rosan, Daniela Padilla-Contreras, Simona Daniela Cavalu, Richard Zsiros, Ioana Maria Borza, Daniela Gitea, Carmen Violeta Iancu, Ertan Yildirim, Murat Aydin, Melek Ekinci, Esma Yigider, Manuel Alexandru Gitea

**Affiliations:** 1 Department of Food Engineering, Faculty of Environmental Protection, University of Oradea, 410048 Oradea, Romania; crosan@uoradea.ro (C.A.R.); ciancu@uoradea.ro (C.V.I.); 2 Doctoral School of Biomedical Science, University of Oradea, 410087 Oradea, Romania; daniela.cavalu@didactic.uoradea.ro (S.D.C.); zsiros.richard@student.uoradea.ro (R.Z.); 3 Laboratory of Physiology and Plant Nutrition for Fruit Trees, Faculty of Agricultural Sciences and Environment, Universidad de La Frontera, Av. Francisco Salazar 01145, Temuco 4811230, Chile; daniela.padilla@ufrontera.cl; 4 Doctoral Program in Science of Natural Resources, Universidad de La Frontera, Av. Francisco Salazar 01145, Temuco 4811230, Chile; 5 Preclinical Sciences Department, Faculty of Medicine and Pharmacy, University of Oradea, 410087 Oradea, Romania; 6 Department of Agriculture, Horticulture, Faculty of Environmental Protection, University of Oradea, 410048 Oradea, Romania; iborza@uoradea.ro (I.M.B.); mgitea@uoradea.ro (M.A.G.); 7 Department of Pharmacy, Faculty of Medicine and Pharmacy, University of Oradea, 410028 Oradea, Romania; dgitea@uoradea.ro; 8 Department of Horticulture, Faculty of Agriculture, Atatürk University, Erzurum 25240, Turkey; ekincim@atauni.edu.tr; 9 Department of Agricultural Biotechnology, Faculty of Agriculture, Atatürk University, Erzurum 25240, Turkey; maydin@atauni.edu.tr (M.A.); esma.yigider@atauni.edu.tr (E.Y.)

**Keywords:** broccoli, selenium nanoparticles, water deficit, oxidative stress mitigation, redox modulation, hormetic response

## Abstract

Drought stress is one of the major constraints limiting crop productivity, primarily through oxidative damage, pigment degradation, and metabolic imbalance. Nanostructured selenium particles (SeNPs) have recently attracted attention for their potential to enhance plant tolerance to abiotic stress. In this study, green-synthesized SeNPs, with a main hydrodynamic size distribution in the range of 90–100 nm, were foliar applied to broccoli (*Brassica oleracea* var. *italica*) plants grown under well-watered (100% water holding capacity) and drought (50% water holding capacity) conditions at concentrations of 0, 10, 20 and 50 ppm. Drought stress significantly decreased chlorophyll a and b, total chlorophyll, and carotenoids, while increasing malondialdehyde (MDA) and proline levels, confirming oxidative stress and membrane damage. SeNPs treatments partially mitigated these effects by enhancing pigment stability, increasing carotenoid content, and reducing both MDA and proline accumulation. Phenolic and flavonoid responses exhibited a dose-dependent pattern with the highest stimulation at 50 ppm under drought and moderate enhancement at 10 ppm under optimal irrigation. Antioxidant capacity assays demonstrated that SeNPs modulate plant redox metabolism, in a context-dependent manner, particularly under water deficit. Peroxidase (POD) activity was also significantly induced under drought stress, mainly at 20 ppm. These results indicate that foliar-applied SeNPs can influence physiological and biochemical responses associated with drought tolerance in broccoli. The observed effects are consistent with nanoparticle–leaf surface interactions contributing to redox regulation and stress adaptation, rather than implying direct nanoparticle internalization.

## 1. Introduction

The intensifying global water crisis and the corresponding rise in drought frequency and severity require novel ways to enhance agricultural resilience. Conventional agricultural methods sometimes fail to alleviate the significant effects of extended water scarcity on crop productivity and quality, necessitating a transition to new approaches [1]. In this context, nanotechnology has emerged as a transformative field, offering novel solutions to enhance agricultural sustainability and productivity, particularly under abiotic stresses such as drought [2]. Specifically, nanoselenium particles (SeNPs), due to their unique physicochemical properties and enhanced bioavailability compared to bulk selenium (Se), present a promising avenue for improving crop tolerance to drought conditions [3]. The unique properties of SeNPs, such as its high surface-area-to-volume ratio and catalytic activity, enable improved nutrient uptake and stress response mechanisms in plants [4].

Compared to ionic selenium, SeNPs are reported to have lower toxicity and a broader effective concentration range, allowing plants to benefit from selenium supplementation without the negative effects linked to ionic forms [5]. Their nanoscale size enables more efficient absorption and distribution within plant tissues, leading to more controlled physiological responses under stress [6]. Instead of completely scavenging reactive oxygen species, SeNPs seem to modulate antioxidant metabolism and redox balance, helping to preserve Reactive Oxygen Species (ROS)-dependent signaling pathways essential for stress adaptation [6,7]. Also, SeNPs can trigger adaptive mechanisms such as improved antioxidant defenses and osmotic regulation, supporting a hormetic, eustress-driven process that ultimately enhances drought tolerance [5,7]. Treatments with SeNPs have also increased the relative water content and levels of photosynthetic pigments in plants under drought stress [8].

This enhanced efficiency allows for reduced application rates while maximizing beneficial outcomes, distinguishing SeNPs from conventional Se applications [9].

Broccoli (*Brassica oleracea* var. *italica*) is a nutritionally valuable cruciferous vegetable, widely cultivated for its high content of vitamins, minerals, and bioactive compounds with antioxidant and anticancer properties [10]. However, its growth and productivity are highly sensitive to abiotic stresses, particularly drought, which is becoming increasingly prevalent due to climate change and limited water resources [11]. Previous studies have shown that broccoli is highly responsive to water deficit, with drought stress negatively affecting its growth, physiological processes, and product quality. This pronounced sensitivity makes broccoli a suitable and informative model for investigating drought tolerance mechanisms in economically important horticultural crops [12,13]. In addition, strengthening drought resilience in broccoli cultivation has clear implications for agricultural sustainability, as improvements in water use efficiency and stress tolerance can contribute to yield stability while reducing irrigation demands under climate change scenarios, especially in water-limited vegetable-growing regions [12,14]. Drought stress negatively affects plant water relations and photosynthetic efficiency, leading to oxidative damage through the overproduction of ROS [15]. As a result, developing sustainable strategies to enhance drought tolerance in broccoli is of both agronomic and environmental importance.

Several crops have been studied for the effects of SeNPs under drought conditions. SeNPs have been shown to improve drought tolerance, growth, and yield in various crops by enhancing antioxidant activity, water retention, and stress resilience. However, foliar application of selenium in combination with silicon (as Se/SiO_2_-NPs) often produces more pronounced protective effects under drought, indicating a synergistic interaction between selenium and silicon in enhancing physiological and biochemical stress responses. For example, in strawberry plants exposed to varying drought levels, treatment with Se/SiO_2_-NPs resulted in greater improvements in photosynthetic pigments, osmolyte accumulation, antioxidant enzyme activity, water use efficiency, and yield attributes compared with plants treated with SeNPs or SiO_2_NPs alone, suggesting enhanced drought tolerance due to the combined action of both elements [16]. In another study [17], the Se nanoparticle foliar spraying, particularly with 10 nm particles, alleviated many of the deleterious effects of drought on pomegranate leaves and fruits. The foliar application of Se nanoparticles during the reproductive stage, particularly at higher concentrations (e.g., 200 mg L^−1^), effectively enhances soybean’s tolerance to drought stress by improving water status, photosynthetic efficiency, antioxidant defense systems, and maintaining cellular integrity [8]. Also, the SeNPs foliar application at 20 mg L^−1^ improves drought tolerance in sorghum by reducing transpiration rate and increasing antioxidant defense system, without causing toxicity to soil, aquatic, and terrestrial organisms [18]. Organic selenium and SeNPs enhance drought stress resistance in pak choi by increasing photosynthetic capacity, maintaining water homeostasis, and enhancing weight through upregulation of metabolic pathways [9]. SeNPs can enhance the nutritional quality and antioxidant capacity of broccoli sprouts. Application of SeNPs to broccoli sprouts increased total biomass at moderate concentrations (10–50 ppm), with no observed toxicity. SeNPs did not significantly affect chlorophyll, carotenoid, or total phenol content, but did alter individual glucosinolate levels and increased antioxidant capacity, especially at higher concentrations (100 ppm) [19]. Broccoli sprouts effectively absorbed SeNPs, suggesting potential for biofortification and improved health benefits [19].

The interaction of nanoparticles with leaves may follow several pathways: (i) cuticular penetration, via aqueous pores on the leaf surface estimated at approximately 2 nm, which favors only very small molecules or ions [20]; (ii) stomatal entry, where size-exclusion limits typically exceed 10 nm and efficient uptake is often reported for particles < 50 nm [21]; and (iii) surface-mediated interactions, in which larger particles remain adsorbed on the cuticle or within stomatal chambers and exert localized effects (hydric microenvironment, light scattering, redox interface) rather than full internalization [22]. Given these mechanisms, the biological effects of SeNPs may derive either from intracellular uptake and redox modulation or from external interactions at the leaf surface.

There is limited direct research on the effects of SeNPs in broccoli under drought conditions. However, studies have explored SeNPs’s impact on broccoli sprouts and its general role in plant stress tolerance. Nanomaterials, including SeNPs, are discussed as promising tools to enhance nutrient uptake and drought resistance in broccoli; however, more targeted research is needed to confirm their specific effects and safety [23]. While SeNPs shows promise for improving nutritional quality and stress tolerance in Brassica crops, there is currently no direct evidence for its effects on broccoli under drought conditions. Research in related species and on broccoli sprouts suggests potential benefits, but further studies are needed to confirm these effects in broccoli facing drought stress.

Therefore, the present study aimed to investigate the potential of green-synthesized SeNPs, obtained using *Petroselinum crispum* (Mill.) Fuss (parsley) extract, in alleviating the adverse effects of drought stress in broccoli. The main objectives were to evaluate the influence of different foliar concentrations of SeNPs on (i) morphological parameters (plant height, stem diameter), (ii) photosynthetic pigment indicators (chlorophyll a, chlorophyll b, total chlorophyll, and carotenoids), and (iii) biochemical and hormonal markers (total phenolics, flavonoids, antioxidant capacity, proline, MDA, IAA (Indole-3-acetic acid), and POD activity) under both well-watered (WW) and drought-stressed conditions (DS). This study provides novel insights into the potential application of plant-mediated SeNPs as an eco-friendly strategy to improve drought tolerance in cruciferous vegetables.

Compared with previous studies that typically focused on isolated biochemical or physiological responses, this work adopts an integrative perspective by simultaneously assessing multiple oxidative stress markers, antioxidant parameters, and hormonal responses. By combining these endpoints, the study provides clearer insights into the hormetic, dose-dependent, and redox-modulating effects of foliar-applied, plant-mediated SeNPs. Overall, this comprehensive evaluation advances the understanding of how green-synthesized nanoselenium contributes to enhanced drought tolerance in cruciferous crops.

## 2. Materials and Methods

### 2.1. Plant Materials and Growth Conditions

The experiment was conducted in a controlled glass greenhouse located within the Interdisciplinary Research Center in Bioeconomy at the Faculty of Environmental Protection, University of Oradea, Romania. Broccoli seeds (*Brassica oleraceae* var. *Italica*, Gentleman, Hybrid F1, produced by Franchi Sementi, (Bergamo, Italy) were sown on 2 June 2023, in elform EPE70 alveolar trays at a depth of 1–1.5 cm, using a commercial sowing substrate (Klasmann TS3 Peat). When the seedlings reached the stage of 2–3 true leaves on 18 June 2023, they were transplanted into VCG10.5 pots (with dimensions of 10.5 cm × 8.2 cm and a volume of 0.46 L). One month after transplantation, the plants were thinned to one seedling per pot and transferred to a VCG19 pot with dimensions of 19 cm × 14.9 cm, a volume of 3 L. The pots were filled with a soil mixture consisting of garden soil and sand in a volumetric ratio of 2:1, with an apparent density of 1.30 gr. cm^3^. The environmental conditions in the greenhouse were maintained at a relative humidity of 60–70%, with day/night temperature ranges of 28–36 °C and 18–21 °C, respectively. Each treatment was repeated five times.

### 2.2. Experimental Design and Treatments

The experiment included eight treatment groups and began on 18 July 2023, arranged in a completely randomized design with five replicates per treatment. The experimental variants are shown in Table 1.

To apply drought stress, the water holding capacity (WHC) of the soil mixture was first determined. FC was determined gravimetrically by saturating soil samples with water, allowing them to drain for 24–48 h, and calculating the water content based on the difference between the moist and oven-dry weights. Well-watered plants were irrigated to maintain 100% WHC. In contrast, drought-stressed plants received 50% of the water volume applied to the well-watered treatments, with adjustments made throughout the experiment based on pot weight and evapotranspiration loss.

One day after the initiation of drought stress, foliar SeNPs treatments were initiated and subsequently applied once per week (total of five applications) for the designated treatments. Foliar treatments were performed using aqueous solutions of SeNPs at concentrations of 10, 20, and 50 ppm. To facilitate droplet spreading and penetration through the leaf cuticle, all SeNPs solutions contained 0.05% (*v*/*v*) Tween 20 (polyoxyethylene (20) sorbitan monolaurate). The selected concentrations (10, 20, 50 ppm) are within the range most commonly tested in plants (5–100 ppm), allowing the assessment of low, intermediate, and relatively high but non-toxic doses. This range was chosen to explore potential hormetic or near-hormetic responses, as supported by previous reports in grapevine, canola, Gerbera, and melon [24,25,26,27], where low-to-moderate doses enhance redox metabolism while higher concentrations tend to lose efficacy or induce mild inhibition. Plants were sprayed with a hand-held atomizer until incipient runoff, following standard foliar application practices used in controlled-environment studies. Although the exact spray volume per plant was not recorded, applications were performed uniformly across all treatments to ensure comparable coverage of the aerial parts. Control plants were treated with distilled water supplemented with the same concentration of Tween 20 to exclude any potential effect of the surfactant itself. The experiment lasted 40 days from the initiation of drought stress. Throughout this period, non-destructive measurements were taken, including leaf chlorophyll content (SPAD), plant height (in mm), and stem diameter (in mm). Foliar spraying was performed weekly, and non-destructive measurements (T1–T5) were recorded immediately prior to each weekly application to ensure consistent timing. At the end of the experiment, leaves were harvested for biochemical analyses. The following parameters were determined: total chlorophyll (Chl) content (spectrophotometric method), total phenolic content (TPC, Folin–Ciocalteu method), total flavonoid content (TFC, aluminum chloride colorimetric method), antioxidant capacity (specify the method used, e.g., DPPH (2,2-Diphenyl-1-Picrylhydrazyl), TEAC (Trolox Equivalent Antioxidant Capacity), FRAP (Ferric Reducing Antioxidant Power), CUPRAC (Cupric Reducing Antioxidant Capacity), proline content, IAA level, MDA content (as a marker of lipid peroxidation), and POD.

**Figure 1:**
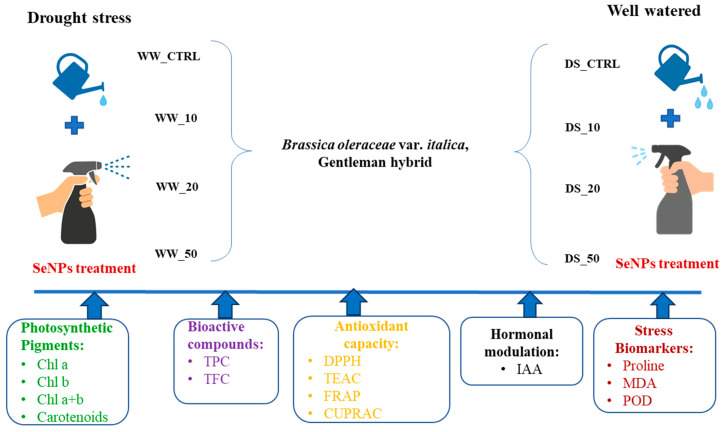
Experimental design of the study and integrated outcome measures. Broccoli plants were grown under two water regimes: well-watered (WW) and drought stress (DS). Within each regime, plants were foliar-sprayed with SeNPs at four concentrations (0, 10, 20, and 50 ppm), using Tween 20 (0.05% *v*/*v*) as a surfactant. Each treatment was replicated in a completely randomized design. The figure summarizes the factorial structure (2 × 4 treatments) used to evaluate the effects of SeNPs on photosynthetic pigment profile, biochemical, and antioxidant responses.

The experimental layout, including treatments, water regimes, and SeNPs concentrations, is illustrated in Figure 1. Plants were subjected to two water regimes (well-watered and drought stress) and treated with SeNPs foliar sprays at 0, 10, 20, and 50 ppm, with each treatment replicated in a completely randomized design.

### 2.3. Green Synthesis of SeNPs

Biosynthesis of SeNPs was performed using the leaves of *Petroselium crispum* (Mill.) Fuss, cultivated in our laboratory, following previously described protocols with minor modifications [24,25,26,27]. In brief, fresh parsley leaves were washed and freeze-dried using the device Freeze dryer Alpha 1–2 Christ (Martin Christ, Osterode am Harz, Germany). Distilled water was added at a ratio 1:10 (*w*/*v*) to rehydrate the leaves, followed by homogenized. After filtration, a Na_2_SeO_3_ solution (10,000 ppm) was added to the plant extract at volume ratios of 1:10 and 1:1 (*v*/*v*) and the reaction mixture was incubated overnight at room temperature until a visible color change to red indicated SeNPs formation. After centrifugation at 5000 rpm for 10 min, the supernatant was removed, and the red SeNPs were washed several times with distilled water, followed by repeated centrifugations (three times), filtrations, and drying. The formation of SeNPs was preliminarily confirmed by UV–Vis spectroscopy in the range of 200–400 nm using a Shimadzu UV-VIS 1700 PharmaSpec spectrophotometer (Shimadzu Corp., Kyoto, Japan), showing a characteristic absorption maximum around 270 nm (Figure S1, Supplementary Materials). The hydrodynamic particle size, size distribution, and zeta potential of SeNPs were determined by dynamic light scattering (DLS) (ZEN 3690, Malvern Instruments Ltd., Malvern, UK) The shape and morphology of Se nanoparticles were further examined by TEM (transmission electron microscopy-Tecnai G2 F30 S-TWIN, FEI Company, Hillsboro, OR, USA)) coupled with Energy Dispersive X-ray Spectroscopy (EDX) for elemental composition analysis.

### 2.4. Non-Destructive Morphophysiological Measurements

Non-destructive morphophysiological measurements were performed on all plants in each treatment group (*n* = 5 plants per treatment) throughout the experiment to monitor growth dynamics and stress responses under different watering and SeNPs treatments.

During the 40-day experimental period, three non-destructive parameters were monitored weekly to evaluate the effects of drought stress and SeNPs treatments on broccoli growth and physiology.

Leaf chlorophyll content was measured using a portable SPAD-502PLUS Digital Chlorophyll Meter (Konica Minolta, Tokyo, Japan). Readings were taken from the fully expanded upper leaves, avoiding the midrib area. For each plant, three measurements were recorded and averaged to obtain a representative value.

Plant height (cm) was measured from the base of the stem at the soil surface to the apical meristem, using a standard ruler.

The stem diameter (mm) was measured at approximately 1 cm above the soil surface using a digital caliper with a 0.01 mm resolution.

### 2.5. Biochemical Analyses

- Total chlorophyll content

Concentrations of chlorophyll *a* (Chl a), chlorophyll *b* (Chl b), and carotenoids were determined spectrophotometrically following the protocol of Nayek et al. [28] with minor modifications. Briefly, 0.5 g of fresh leaf tissue was homogenized at 15,000 rpm for 300 s using an Ultra-Turrax homogenizer (SilentCrusher M, Heidolph Instruments GmbH & Co., Schwabach, Germany) in 10 mL of ice-cold 95% ethanol. The homogenates were centrifuged at 12,000 rpm for 10 min at 4 °C. An aliquot of 0.5 mL from the resulting supernatant was diluted with 4.5 mL of 95% cold ethanol. Absorbance was measured at 664 nm, 649 nm, and 470 nm using a Shimadzu UV-1240 mini UV–Vis spectrophotometer (Shimadzu, Kyoto, Japan). Pigment concentrations (µg/mL) were calculated using the specific equations provided by Nayek et al. [28], and the final results were expressed in µg/mL.

- Total phenolic content

The total phenolic content (TPC) in broccoli leaves was determined using the Folin–Ciocalteu colorimetric method with slight modifications, as previously described by Vicas et al. [19]. Briefly, 100 µL of each ethanol extract was mixed with 1.7 mL of distilled water, followed by the addition of 200 µL of freshly prepared Folin–Ciocalteu reagent (diluted 1:10, *v*/*v*) and 7.5% sodium carbonate (Na_2_CO_3_) solution. The mixture was incubated in the dark at room temperature for 2 h to allow color development. Absorbance was measured at 765 nm using a Shimadzu UV-1240 mini UV–Vis spectrophotometer (Shimadzu, Kyoto, Japan). Gallic acid was used as a standard, and the results were expressed as mg GAE/g fresh weight (FW).

- Total flavonoid content

The total flavonoid content (TFC) of broccoli leaves was determined using the aluminum chloride (AlCl_3_) colorimetric method, as previously described [29], with minor modifications. Briefly, 1 mL of ethanol leaf extract was transferred into a 10 mL volumetric flask containing 4 mL of distilled water. Then, 300 µL of 5% sodium nitrite (NaNO_2_) was added. After 5 min, 300 µL of 10% AlCl_3_ solution was added, followed by 2 mL of 1 M sodium hydroxide (NaOH) after another 6 min. The volume was adjusted to 10 mL with distilled water, and the mixture was then mixed thoroughly. Optical density was recorded at 510 nm against a reagent blank using a Shimadzu UV-1240 mini UV–Vis spectrophotometer (Shimadzu Corporation, Kyoto, Japan). Quercetin was used as the standard, and results were expressed as milligrams of quercetin equivalent (mg QE)/g FW.

### 2.6. Antioxidant Capacity

- DPPH assay

The radical scavenging capacity of ethanol leaf extracts was assessed using the DPPH assay, as described by Brand-Williams et al. with minor modifications [30,31]. A volume of 100 µL of each extract was mixed with 2.8 mL of freshly prepared 80 µM DPPH solution in methanol. The mixture was incubated for exactly 30 min in the dark at room temperature. After incubation, the absorbance was measured at 517 nm using a Shimadzu UV-1240 mini-UV–Vis spectrophotometer. The radical scavenging activity (*RSA*) was calculated using Equation (1):

(1) $$\mathrm{RSA}\;(\%)\;=\frac{\left(A_{0}-A_{1}\right)}{A_{0}}\cdot 100,$$

where A_0_ is the absorbance of the DPPH solution without extract (control) and A_1_ is the absorbance in the presence of the extract.

Results were expressed as µmol Trolox equivalents (TE)/g FW, using a Trolox calibration curve.

- FRAP assay

The FRAP value of broccoli leaf extracts was determined following the protocol described by Vicas et al. [31,32], with slight modifications. Briefly, 100 µL of ethanol extract was mixed with 2.0 mL of distilled water and 500 µL of freshly prepared FRAP working solution. The FRAP reagent was prepared by mixing 300 mM acetate buffer (pH 3.6), 10 mM 2,4,6-tripyridyl-s-triazine (TPTZ) solution in 40 mM HCl, and 20 mM FeCl_3_·6H_2_O in a 10:1:1 (*v*/*v*/*v*) ratio. The reaction mixture was incubated in the dark at room temperature for 60 min. Absorbance was read at 595 nm using a Shimadzu UV-1240 mini UV–Vis spectrophotometer. Data were normalized to fresh weight and expressed as µmol TE/g FW, based on a Trolox standard calibration curve.

- TEAC Assay

The antioxidant capacity of broccoli leaf extracts was also evaluated using the TEAC assay, based on the ABTS•^+^ (2,2′-azinobis-(3-ethylbenzothiazoline-6-sulfonic acid)) radical cation decolorization method, as described by Vicas et al. [19], with slight modifications. The ABTS•^+^ radical cation was generated by mixing 7 mM ABTS stock solution with 2.45 mM potassium persulfate and allowing the mixture to react in the dark at room temperature for 12 h. Before use, the ABTS•^+^ solution was diluted with ethanol to obtain an absorbance of 0.70 ± 0.02 at 734 nm. For the assay, 100 µL of leaf extract was added to 2.9 mL of the diluted ABTS•^+^ solution, and the absorbance was recorded at 734 nm after a fixed incubation time using a Shimadzu UV-1240 mini-UV–Vis spectrophotometer. Quantification was performed based on a calibration curve constructed with Trolox as the reference standard and expressed as µmol TE/g FW.

- CUPRAC assay

The cupric reducing antioxidant capacity of broccoli leaf extracts was determined according to the method described by Miere (Groza) et al. [31], with slight modifications. The reaction mixture consisted of 1 mL of 10^−2^ M copper (II) chloride solution, 1 mL of 7.5 × 10^−3^ M neocuproine (2,9-dimethyl-1,10-phenanthroline) alcoholic solution, 1 mL of ammonium acetate buffer (pH 7.0), and 100 µL of ethanol leaf extract. The final volume was adjusted to 4.1 mL with distilled water. The mixture was incubated for 30 min at room temperature. After incubation, absorbance was measured at 450 nm using a Shimadzu UV-1240 mini UV–Vis spectrophotometer. Antioxidant capacity was expressed as µmol TE/g FW, based on a Trolox calibration curve.

### 2.7. Hormonal Modulation

- Indole-3-Acetic Acid (IAA) Content

The concentration of IAA in broccoli leaves was determined using the Salkowski reagent method. Briefly, 100 mg of fresh leaf tissue was homogenized in 1.7 mL of cold phosphate buffer (pH 7.0) using a mortar and pestle. The homogenate was centrifuged at 10,000 rpm for 10 min at room temperature. A volume of 1.2 mL of the resulting supernatant was mixed with 1.2 mL of phosphate buffer. Then, 1 mL of this dilution was combined with 2 mL of Salkowski reagent (prepared as described in the literature [33]. 2 mL of 0.5 M FeCl_3_ with 49 mL water, and 49 mL of 70% perchloric acid, and incubated in the dark at room temperature for 25 min. After incubation, the absorbance was measured at 530 nm using a Shimadzu UV-1240 mini-UV–Vis spectrophotometer. IAA concentration was calculated from a standard curve prepared using known concentrations of indole-3-acetic acid and subsequently converted to molar units using the molecular weight of IAA (175.18 g/mol). Final results were expressed as nmol/g FW.

### 2.8. Markers of Drought Stress

- Proline content

The free proline content in broccoli leaves was determined according to the method of Bates et al., with minor modifications as described by Abrahám et al. [34,35]. Briefly, 0.5 g of frozen leaf tissue was homogenized using a chilled mortar and pestle with 10 mL of 3% sulfosalicylic acid. The homogenate was centrifuged at 5000 rpm for 5 min at room temperature (NÜVE NF 200, Ankara, Turkey). The resulting supernatant was mixed in equal volumes (1:1:1, *v*/*v*/*v*) with acid ninhydrin reagent and glacial acetic acid. The reaction mixture was incubated at 96 °C for 1 h and then immediately transferred onto ice to stop the reaction. The chromophore formed was extracted with 4 mL of toluene by vigorous vortexing for 15–20 s. The absorbance of the toluene phase was measured at 520 nm using toluene as a blank, with a Shimadzu UV-1240 mini-UV–Vis spectrophotometer. Proline concentration was calculated from a standard curve prepared with known concentrations of L-proline (5–100 µg/mL), converted to micromoles using the molecular weight of proline (115.5 g/mol), and finally expressed as µmol/g FW.

- Peroxidase (POD) Activity

Peroxidase (POD) activity in broccoli leaves was determined based on the method described by Kim and Yoo (1996) [36], with slight modifications. Fresh leaf samples (0.1 g) were homogenized using a mortar and pestle in 1 mL of 0.1 M phosphate buffer (pH 7.0) for 5 min on ice. The homogenate was centrifuged at 14,000 rpm for 5 min at 4 °C, and the supernatant was used as the enzyme extract. The reaction mixture (3.05 mL total volume) contained the following:1 mL of 0.1 M phosphate buffer (pH 7.0), 1 mL of 1.5 mM guaiacol (2-methoxyphenol), 1 mL of 3 mM hydrogen peroxide (H_2_O_2_), and 50 µL of enzyme extract. The reaction was carried out at 30 °C, and the increase in absorbance due to the formation of tetraguaiacol was recorded at 470 nm using a Shimadzu UV-1240 mini-UV–Vis spectrophotometer. Absorbance was measured at time zero (A_0_) and after 1 min (A_1_). Enzyme activity was calculated using Equation (2):

(2) $$\mathrm{POD}\;\mathrm{activity}\;\left(U/\mathrm{mL}\right)=\frac{\left(A_{1}-A_{0}\right)\cdot V_{t}}{\varepsilon \cdot l\cdot V_{s}},$$

where ΔA (A_1_ − A_0_) is the change in absorbance per minute, V_t_ is the total reaction volume (3.05 mL), vs. is the volume of enzyme extract (0.05 mL), and ϵ is the molar extinction coefficient of tetraguaiacol at 470 nm (26.6 mM^−1^cm^−1^).

Final enzyme activity was initially calculated and expressed in units per milliliter (U/mL), where one unit corresponds to the amount of enzyme that oxidizes 1 µmol of guaiacol per minute under the assay conditions. For comparison among treatments, peroxidase activity was further expressed on a fresh weight basis (U/g FW) by normalizing the activity to the mass of fresh tissue used for extraction, taking into account the total extraction volume and any dilution factors.

- Malondialdehyde (MDA) Content

The level of lipid peroxidation in broccoli leaves was estimated by measuring MDA content, using a thiobarbituric acid reaction, with slight modifications from the standard protocol [37]. Fresh leaf samples (500 mg) were homogenized in 4 mL of 1% trichloroacetic acid. The homogenate was centrifuged at 5000 rpm for 20 min at room temperature. The supernatant was collected and centrifuged again under the same conditions to remove any residual debris. Next, 1.5 mL of the clarified supernatant was mixed with 1.5 mL of TBA reagent (prepared by dissolving 150 mg of TBA in 30 mL of 20% TCA). The mixture was incubated at 95 °C for 30 min in a water bath, followed by rapid cooling in an ice bath. The samples were then centrifuged at 5000 rpm for another 20 min. The absorbance of the resulting solution was recorded at 440, 532, and 600 nm using a Shimadzu UV-1240 mini-UV–Vis spectrophotometer. Results were expressed as µmol MDA/g FW.

### 2.9. Leaf Mass per Area (LMA)

Frozen leaf fragments were briefly thawed and photographed on a flat white background next to a 1 cm^2^ calibration square. Leaf area (A, cm^2^) was quantified in ImageJ (v.1.54m, National Institutes of Health, Bethesda, MD, USA) by setting the scale using the 1 cm^2^ reference square, applying thresholding, and analyzing particles. The same leaf fragments were then dried at 65 °C to constant weight, cooled in a desiccator, and weighed to obtain dry mass (DW, mg). LMA was calculated as DW/A (mg·cm^2^). Midribs were included consistently across treatments. This method is suitable for frozen samples, as LMA depends on dry mass and lamina area, which remain stable after freezing [38].

### 2.10. Statistical Analysis

For the analysis of plant height (cm), stem diameter (mm), and SPAD index, the treatment factor (eight applications) was treated as an eight-level between-subjects factor, while time was treated as a five-level within-subjects (repeated-measures) factor. Since repeated measurements were collected from five plants within each treatment group, all variables were analyzed using a two-way mixed-design ANOVA (Repeated Measures ANOVA). The assumption of sphericity for repeated measures was assessed using Mauchly’s test; if violated, the Greenhouse–Geisser correction was applied. Significant main effects or Treatment × Time interactions were further evaluated using Tukey’s HSD pairwise comparisons. For morphological measurements (height, stem diameter, SPAD), five biological replicates per treatment (*n* = 5) were used because these variables were measured nondestructively on all plants. For biochemical and antioxidant assays, three biological replicates (*n* = 3) were used due to destructive sampling requirements, with each replicate consisting of pooled leaf tissue to ensure sample homogeneity.

For the remaining variables, statistical differences among the eight treatment groups were evaluated using a one-way ANOVA, followed by Tukey’s HSD post hoc test (*p* < 0.05). Data were expressed as mean ± standard deviation (SD) and treatments not sharing the same letter were considered significantly different. All statistical analyses were performed using JASP software (v. 0,95,1). Relative values for each parameter were calculated as the ratio between the treated sample and the corresponding control within each water regime (WW or DS). A reference value of 1.0 was used to indicate no change relative to the control. To enable an intuitive comparison of treatment effects, a heatmap was constructed using a normalized color scale ranging from 0.5 to 2.0. In this visualization, values <1.0 (blue shades) represent decreases relative to the control, whereas values >1.0 (red shades) indicate increases. This visualization allows a direct assessment of the dose-dependent responses under both well-watered and drought-stressed conditions.

## 3. Results

### 3.1. The Morphology and Size of SeNPs Used in Treatment

**Figure 2:**
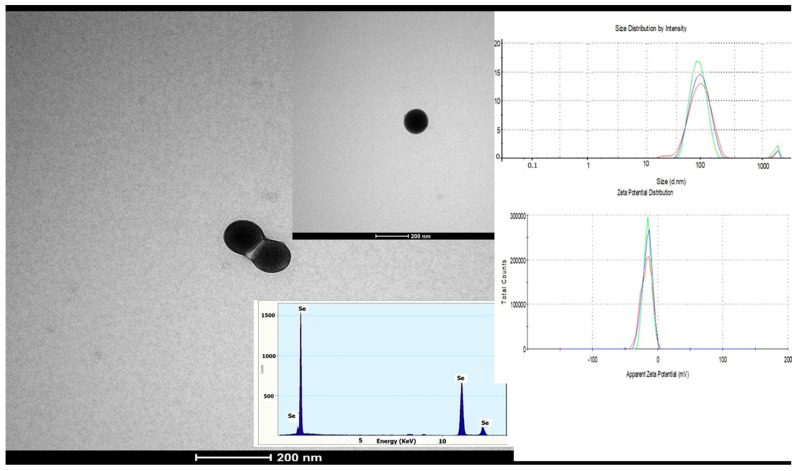
Transmission electron microscopy (TEM) images of SeNPs, and corresponding EDX spectrum (inset), Particle size distribution by intensity, and Zeta potential distribution measurement by DLS. Colored lines represent different SeNPs concentrations (10, 20, and 50 ppm).

The formation of SeNPs was confirmed by dynamic light scattering (DLS), which revealed a narrow particle size distribution with a main population in the range of 90–100 nm, as shown in Figure 2 (inset). The intensity-weighted size distribution exhibited a single dominant peak, indicating a relatively homogeneous nanoparticle population. The Zeta potential indicated negative value of approximately −24 mV, suggesting good colloidal stability and low aggregation tendency. The morphology of SeNPs as evidenced by TEM, revealed predominantly spherical particles, with an average diameter of approximately 110 nm. Figure 2 presents representative TEM micrographs of SeNPs, along with the corresponding EDX spectrum confirming the elemental composition and the DLS size and zeta potential distributions.

### 3.2. The Effects of Drought Stress and SeNPs Treatments on Broccoli Growth and Chlorophyll Content (SPAD)

Repeated-measures ANOVA showed that, for plant height, the main effect of Treatment was marginal after correcting for sphericity (Greenhouse–Geisser: MS = 2914.94, F = 2.87, *p* = 0.0946), while the effect of Time was strongly significant (MS = 19,880.04, F = 74.23, *p* < 0.0001), indicating substantial changes throughout the experimental period. The Treatment × Time interaction remained significant under the Greenhouse–Geisser correction (MS = 1506.30, F = 3.73, *p* = 0.042), suggesting treatments differently affected height over time. For stem diameter, the Treatment effect was not significant across corrections (Greenhouse–Geisser: MS = 6.23, F = 1.27, *p* = 0.332), but the effect of Time was highly significant (MS = 83.02, F = 123.96, *p* < 0.0001), showing consistent increases in stem diameter across weeks. The Treatment × Time interaction was non-significant (Greenhouse–Geisser: MS = 5.67, F = 3.27, *p* = 0.064), indicating that treatment effects across time points approached significance. For the SPAD index, the main effect of Treatment was not significant (Greenhouse–Geisser: MS = 507.63, F = 2.92, *p* = 0.116), whereas Time had a strong effect (MS = 5917.79, F = 157.61, *p* < 0.0001). The Treatment × Time interaction was significant before correction (MS = 26.16, F = 2.16, *p* = 0.0025) but not after applying the Greenhouse–Geisser adjustment (MS = 259.65, F = 2.16, *p* = 0.152).

To further characterize growth patterns, plant height was evaluated at five weekly intervals (Table 2). Plant height was significantly influenced by both water regime and SeNPs application. Under well-watered conditions, control plants (WW_0) exhibited the highest and uniform growth, reaching 135.20 ± 13.18 cm at the final time point. Compared to the initial measurement, this represents an overall increase of ~59%. In contrast, SeNPs-treated plants (10–50 ppm) under well-watered conditions displayed a similar growth trend but remained shorter than the control throughout the experiment, with final heights ranging from 107.50 ± 11.15 cm (WW_20) to 123.20± 2.28 cm (WW_10). Under drought stress (DS), plant height was consistently reduced. The untreated control (DS_0) reached only 103.67 ± 19.13 cm at the end of the experiment, which corresponds to an ~30% reduction compared to the well-watered control. Growth dynamics under drought were slower, with lower percentage increases between intervals. Among SeNPs treatments, DS_10 and DS_50 plants reached slightly higher final values (110.60 ± 15.53 cm and 112.40 ± 17.92 cm, respectively), suggesting a partial alleviation of growth inhibition. By contrast, DS_20 showed the weakest performance (102.8 ± 9.83 cm), even lower than the drought control. Overall, while SeNPs applications under well-watered conditions did not promote plant height, under drought stress, the 10 and 50 ppm doses mitigated growth reduction, pointing to a dose-dependent protective effect.

Under well-watered conditions, SeNPs treatments (10–50 ppm) resulted in reduced stem diameter compared to WW control (Table 2), particularly at later time points. The most pronounced decreases were observed in WW_20 and WW_50. Under WW conditions, SeNPs application produced minor effects on stem thickness, with reductions of approximately 3–18% relative to WW_0 at 10–50 ppm. By contrast, under DS conditions, SeNPs treatments partially mitigated stem shrinkage. At the final time point, DS_10 and DS_20 showed only 4–7% reductions compared with DS_0, while DS_50 remained close to control levels. This trend indicates that moderate foliar SeNPs doses (10–20 ppm) improved stem robustness and reduced drought-induced thinning, likely through enhanced physiological performance and stress resilience under drought conditions. SPAD values (Table 2) increased steadily throughout the experiment, reflecting the progressive accumulation of chlorophyll. Under well-watered conditions, moderate doses of SeNPs (10–20 ppm) followed growth trajectories similar to the control, whereas WW_50 showed the lowest SPAD value (61.42 ± 5.37), suggesting a slight inhibitory effect of higher SeNPs on pigment retention. Under DS conditions, chlorophyll content was generally lower (DS_0 = 70.0 ± 8.09, approximatively 5% lower than WW_0). The gradual increase observed after Time 3 in all drought treatments under SeNPs treatments indicates a partial recovery of chlorophyll biosynthesis, supporting a protective role of foliar SeNPs, especially at moderate doses.

### 3.3. Biochemical Parameters of Broccoli Under Drought and Nanoparticles Treatments

#### 3.3.1. Photosynthetic Pigments

Drought stress had a strong negative impact on photosynthetic pigments. Both Chl a and Chl b decreased significantly compared to well-watered plants, resulting in reduced total chlorophyll content (Table 3). SeNPs treatments alleviated these effects, with 20 and 50 ppm restoring pigment levels closer to those of the WW_0 sample. The Chl a/b ratio also differed between treatments: in the drought control, it was reduced due to a stronger decline in Chl a, whereas SeNPs application increased the ratio under drought, in some cases above well-watered plants. Carotenoids followed a similar pattern, with drought controls showing the lowest values and SeNPs treatments (particularly 10 and 20 ppm) increasing carotenoid levels under stress (Table 3).

In addition to individual pigment contents, the ratio of total chlorophyll to carotenoids (Total Chl/CARs) was significantly affected by both drought stress and SeNPs application (Table 3). Drought-stressed control plants exhibited a high Total Chl/CARs ratio, reflecting an imbalance between chlorophyll degradation and carotenoid depletion under severe stress conditions. Foliar application of SeNPs under drought stress significantly affected the Total Chl/CARs ratio (Table 3). The lowest ratio was recorded at 10 ppm, indicating a strong stimulation of carotenoid accumulation and enhanced photoprotective responses. At higher concentrations (20 and 50 ppm), the ratio increased moderately but remained significantly lower than in drought-stressed control plants. This non-linear, dose-dependent pattern reflects a hormetic response, in which low SeNPs doses induce maximal photoprotection, while higher doses maintain pigment balance and delay stress-induced aging of the photosynthetic apparatus. Under well-watered conditions, SeNPs treatments, especially at higher concentrations, increased the Total Chl/CARs ratio, suggesting improved chlorophyll stability and photosynthetic capacity in the absence of water limitation.

#### 3.3.2. Bioactive Compounds and Antioxidant Capacity

Under drought stress, SeNPs application at 10 and 20 ppm did not result in significant changes in total phenolics compared to drought-stressed control plants (Table 3). At 50 ppm, however, TPC increased significantly by more than 70% relative to the control. Under well-watered conditions, 10 ppm SeNPs slightly increased TPC in a non-significant manner, whereas 20 ppm resulted in the lowest value, with a reduction of more than 50%. At 50 ppm, TPC levels decreased again, though not significantly compared to the control and WW_10 (Table 4).

Under drought stress, SeNPs treatments showed a divergent response depending on the dose in the case of total flavonoids. Compared to the drought control, 10 and 20 ppm significantly reduced TFC, whereas 50 ppm significantly increased it, reaching values higher than those of the well-watered control (Table 3). Under well-watered conditions, SeNPs at 10 and 20 ppm significantly enhanced flavonoid levels compared to the control, while at 50 ppm flavonoids decreased back to control levels (Table 3).

The antioxidant activity, evaluated by DPPH, FRAP, TEAC, and CUPRAC assays, showed strong dependence on both water regime and SeNPs dose (Table 3). Under drought stress, SeNPs significantly increased DPPH radical scavenging activity, with the highest values at 10 and 50 ppm, while 20 ppm produced a moderate rise compared to the control (DS_0). FRAP was maximized at 10 ppm and remained higher at 50 ppm, but not at 20 ppm in both WW and DS conditions. TEAC also increased significantly at all SeNPs doses under drought, reaching maximum values at 10 ppm. Similarly, CUPRAC values rose in all drought-stressed SeNPs treatments, with the strongest effects at 10 and 50 ppm. In contrast, under well-watered conditions, DPPH and TEAC activities were slightly improved at 10 ppm but markedly decreased at 20–50 ppm. CUPRAC followed the same pattern, with a mild increase at 10 ppm but reductions at higher concentrations. CUPRAC FRAP values remained relatively unchanged across treatments under well-watered conditions.

#### 3.3.3. Markers of Abiotic Factor Stress

Under drought, SeNPs application reduced proline levels compared to the untreated drought control (DS_0). The reduction was strongest at 10 ppm (−28%), followed by 20 ppm (−16%) and 50 ppm (−21%). Under well-watered conditions, the control (WW_0) had much lower proline than the drought control. SeNPs increased proline accumulation by +115% at 10 ppm and +77% at 20 ppm, whereas 50 ppm slightly decreased it (−7%) relative to WW_0. (Table 5).

Malondialdehyde (MDA) content, an indicator of lipid peroxidation, was significantly affected by both water regime and SeNPs application (Table 4). Drought stress sharply elevated MDA in the control (DS_0). SeNPs reduced lipid peroxidation by −27% at 10 ppm, −41% at 20 ppm, and −42% at 50 ppm. Under well-watered conditions, SeNPs caused smaller reductions in MDA, ranging from −3% (10 ppm) to −25% (20 ppm), with 50 ppm showing a similar decline (−22%).

Proline content was strongly affected by water regime and SeNPs treatment (Table 4). In the absence of SeNPs, drought markedly increased proline from 1.32 µmol/g FW in WW_0 to 3.83 µmol/g FW in DS_0, confirming its role as an osmotic adjustment metabolite under water deficit. Under drought, foliar SeNPs partially modulated this response. Proline decreased to 2.75 µmol/g FW at 10 ppm, increased at 20 ppm, and reached 3.03 µmol/g FW at 50 ppm. Under well-watered conditions, proline remained lower than in droughted plants but showed a slight increase at 10 and 20 ppm (2.84 and 2.34 µmol/g FW, respectively) compared with WW_0 and WW_50. According to Tukey’s HSD (*p* < 0.05), DS_0 exhibited the highest proline levels, WW_0 and WW_50 the lowest, whereas the remaining treatments formed intermediate groups.

Under drought stress, SeNPs significantly increased POD activity compared to the untreated control (DS_0). At 10 ppm, activity rose by ~36%, while the 20 ppm treatment almost doubled POD levels (+95%). At 50 ppm, activity remained elevated (+30% compared to DS_0), though lower than at 20 ppm. Under well-watered conditions, the control (WW_0) exhibited the lowest POD activity. SeNPs application increased POD by ~48% at 10 ppm, ~66% at 20 ppm, and ~58% at 50 ppm, although these increases were still below the levels observed in drought-stressed plants.

#### 3.3.4. Hormonal Modulation (IAA)

Under drought, SeNPs significantly stimulated IAA accumulation relative to DS_0, with increases of +40% at 10 ppm, +47% at 20 ppm, and +14% at 50 ppm. In contrast, under well-watered conditions SeNPs generally suppressed IAA. Compared to WW_0, levels decreased by −25% at 10 ppm, −15% at 20 ppm, and −15% at 50 ppm (Table 4).

#### 3.3.5. Leaf Mass per Area

**Figure 3:**
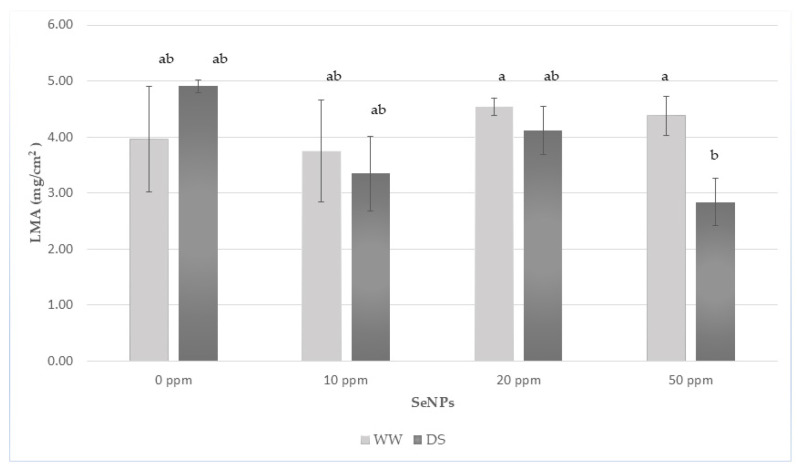
Leaf mass per area (LMA) in broccoli plants subjected to well-watered (WW) and drought-stressed (DS) conditions and foliar application of SeNPs at 0, 10, 20, and 50 ppm. Bars represent mean ± SD (*n* = 3). Different letters indicate significant differences among treatments according to Tukey’s HSD test (*p* < 0.05).

Leaf mass per area (LMA) showed differential responses to drought and NSe application (Figure 3). In control plants (WW_0 vs. DS_0), drought increased LMA (from 3.96 to 4.15 mg·cm^2^), indicating a typical structural thickening of leaves under water deficit. Under well-watered conditions, LMA values ranged between 3.75 and 4.54 mg·cm^2^, with no significant differences among treatments.

Under drought, NSe induced dose-dependent response to SeNPs (Figure 3). Plants treated with 20 ppm maintained relatively high LMA values (4.11 mg·cm^2^), whereas 10 ppm resulted in a moderate decrease (3.48 mg·cm^2^). The lowest LMA was observed at 50 ppm (2.84 mg·cm^2^). According to Tukey’s HSD (*p* < 0.05), only DS_50 differed significantly from WW_20 and WW_50, while the remaining treatments formed a statistically homogeneous group. Overall, drought tended to increase leaf structural investment, while the highest NSe dose reduced this response, resulting in a noticeably thinner and less dense leaf structure under stress.

**Figure 4:**
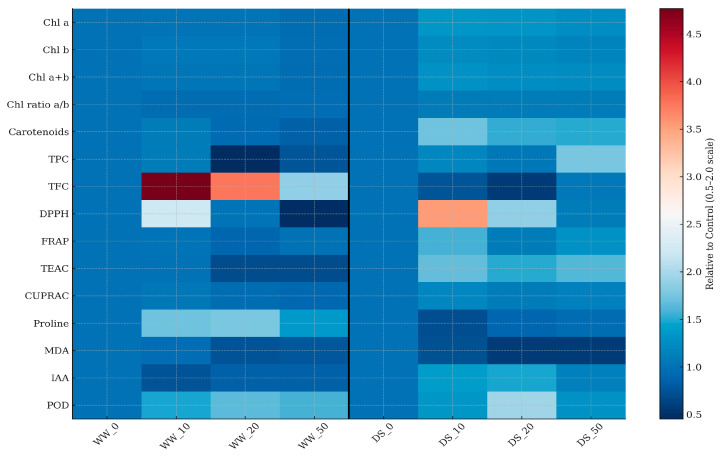
Heatmap of relative responses of broccoli to SeNPs (10–50 ppm) under well-watered (WW) and drought stress (DS) conditions. Values are expressed relative to controls (WW_0, DS_0), with blue indicating decreases (<1) and red increases (>1).

To better visualize these integrated responses, a heatmap of relative values across treatments was constructed (Figure 4). The map highlights the hormetic and context-dependent behavior of SeNPs: under drought stress, most parameters associated with oxidative stress (MDA, proline) appear in the blue zone (<1), indicating attenuation, whereas pigments, phenolics, and antioxidant activities shift toward the red zone (>1), denoting stimulation. Conversely, under well-watered conditions, only low-dose SeNPs (10 ppm) produced red signals in phenolics and flavonoids, consistent with a mild eustress effect, while higher doses shifted many parameters into the blue zone, reflecting suppression. This visualization supports the conclusion that nanoselenium simultaneously enhances protective pathways under drought while displaying dose-dependent inhibition under optimal growth conditions.

## 4. Discussion

The present study aimed to evaluate whether SeNPs can mitigate the detrimental effects of drought stress on broccoli growth, photosynthetic pigments, antioxidant compounds, and stress-related biochemical markers. As expected, drought stress negatively affected plant growth and pigment content in our experiment, confirming the strong sensitivity of broccoli to water deficit. Consistent with previous reports on selenium nanoparticles applied to different crop species, our results indicate that SeNPs can contribute to improved plant performance under abiotic stress conditions through the modulation of physiological and biochemical responses, including the activation of antioxidant defenses and the maintenance of redox homeostasis [24,25,26,27]. Compared to other selenium-based treatments reported in the literature, including bulk or ionic selenium forms, SeNPs appear to be associated with a broader and more coordinated influence on plant stress responses. While conventional selenium forms often affect specific physiological processes, SeNPs act as multifunctional regulators by simultaneously fine-tuning enzymatic and non-enzymatic antioxidant systems, preserving photosynthetic pigments, and maintaining signaling pathways involved in stress adaptation [8,24]. This integrated mode of action supports the concept that selenium nanostructures provide enhanced efficacy at lower doses, contributing to improved stress resilience without inducing toxicity. Such multifunctional behavior distinguishes SeNPs from other selenium formulations and underscores their potential as effective redox modulators under drought conditions [39].

The observed decrease in chlorophyll under drought conditions in our broccoli plants confirms the degradation of photosynthetic complexes and the damage to the photosynthetic apparatus, a well-known response to water deficit [40]. Importantly, SeNPs application partially restored pigment levels, suggesting a protective role against pigment degradation and stabilization of photosystems. The reversal in the Chl a/b ratio under SeNPs treatment was particularly noteworthy, as it indicates preferential protection of Chl a and reaction center chlorophylls, possibly through improved functional stability of photosystem I (PS I) and II (PS II) core complexes, associated with enhancement antioxidant protection. Such adjustments likely contributed to maintaining electron transport and delaying drought-induced senescence. Carotenoids also followed a clear protective trend. In our study, their accumulation under SeNPs was sometimes even above WW control, highlighting their dual role as accessory pigments and photoprotective antioxidants. This pattern may reflect both the stimulation of carotenoid biosynthesis and the protection of biosynthetic enzymes against oxidative inactivation. By enhancing photoprotection, carotenoids help the photosynthetic apparatus cope with drought-induced oxidative stress. Similar protective roles of Se in maintaining chlorophyll pigments and stimulating carotenoid accumulation under abiotic stress have been documented in wheat, rice, and other crops [23,41,42]. In addition to changes in individual pigment contents, the ratio of total chlorophyll to carotenoids (Total Chl/CARs) provides integrative information on the functional status and aging of the photosynthetic apparatus under stress conditions. Under drought stress, this ratio is often reduced because chlorophylls (a + b) are more sensitive to water deficit, undergoing inhibited biosynthesis and accelerated degradation, whereas carotenoids, which play key photoprotective and antioxidant roles, are relatively preserved or decline more slowly.

In the present study, foliar application of SeNPs markedly modulated the Total Chl/CARs ratio under drought conditions, with the lowest values observed at low SeNPs concentration (10 ppm), followed by a moderate increase at higher doses (20 and 50 ppm). This non-linear response reflects a hormetic adjustment, in which enhanced carotenoid accumulation at low SeNPs doses strengthens photoprotection, while higher concentrations maintain pigment balance and photosystem stability. Although hormesis is inferred from the observed dose–response patterns, further targeted studies would be required to confirm the underlying signaling mechanisms. Similar drought-induced reductions in the chlorophyll/carotenoids ratio have been reported in a range of species, including grapevine [43], herbaceous plants [44], and conifers [45], supporting the use of this ratio as a sensitive biochemical indicator of drought tolerance and stress-induced aging of the photosynthetic apparatus.

The relatively low chlorophyll a/b ratios (<2) observed in this study, compared with typical heliophytic species, can be explained by the combined effects of moderate drought stress, nanoselenium application, and greenhouse growth conditions characterized by diffuse natural light. Such conditions favor an increased proportion of chlorophyll b, reflecting a photochemical acclimation strategy aimed at optimizing light absorption and energy distribution under stress [46]. The protective role of SeNPs in maintaining pigment integrity under drought stress has been previously reported [8,9]. In addition, SeNPs exert antioxidant effects on the photosynthetic apparatus, contributing to the limitation of oxidative damage to thylakoid membranes and inhibiting stress-induced pigment degradation [8,9]. This mechanism is consistent with the relatively high Total chlorophyll/carotenoids ratios (>5) recorded in our experiment and the absence of clear senescence symptoms even under drought stress. Moreover, at the time of sampling, broccoli plants were still at an active vegetative stage, and fully expanded but physiologically young leaves were analyzed, a developmental context known to favor pigment stability and lower chlorophyll a/b ratios. Together, these factors indicate that the observed pigment composition reflects adaptive acclimation of the photosynthetic apparatus. While the absolute chlorophyll a/b values reported here differ from those typically cited for field-grown heliophytic crops, they are internally consistent across treatments and are interpreted within the specific experimental context of greenhouse cultivation, vegetative developmental stage, and nanoselenium application, rather than as universal reference values.

Mechanistically, the increase in chlorophyll content under drought conditions with SeNPs supplementation can be explained by multiple interconnected processes: (i) alleviation of oxidative stress, (ii) preservation of photosynthetic efficiency, and (iii) improved osmotic adjustment and nutrient uptake [47]. Our biochemical data reinforce this view, indicating that SeNPs limited the accumulation of H_2_O_2_ and MDA while preserving thylakoid integrity and pigment–protein complexes, thereby favoring pigment stability under drought stress [24,48,49]. Moreover, SeNPs have been reported to be associated with the upregulation of pigment-binding proteins such as Lhca and Lhcb families, crucial for chlorophyll biosynthesis and light harvesting [9]. By maintaining PSII/PSI performance, Se or SeNPs reduces stress signaling pathways that normally activate chlorophyll-degradation enzymes [8,50]. Indeed, Se treatments in other species were shown to preserve chloroplast ultrastructure and pigment pools, while limiting the activity of chlorophyllase and PAO (pheophorbide a oxygenase), two key enzymes in the PAO/phyllobilin pathway [51]. This mechanism is consistent with the slower chlorophyll breakdown observed in our study.

It is also important to consider the dose–response aspect. Although SeNPs exhibit dose-dependent physiological effects, excessive concentrations may still interfere with chlorophyll biosynthesis, particularly at the 5-aminolevulinic acid (ALA) step, leading to reduced pigment precursors [52]. This dose-dependent behavior may explain why low to moderate doses are beneficial under drought, while excessive supplementation could be detrimental.

Beyond pigments, SeNPs also modulate secondary metabolism. By stabilizing chlorophylls and carotenoids and simultaneously fine-tuning phenolic and flavonoid biosynthesis, SeNPs act as a redox modulator in a dose- and environment-dependent manner, linking pigment protection with the reinforcement of antioxidant defenses.

These findings reveal a context-dependent, hormetic modulation of the phenylpropanoid pathway by SeNPs. Under drought conditions, baseline ROS levels are already high and endogenously activate the phenylpropanoid pathway. Thus, SeNPs at 10–20 ppm produced little extra stimulation of total phenolics (TPC), whereas 50 ppm markedly boosted TPC, consistent with reports that Se/SeNPs further activate phenylpropanoid metabolism to reinforce antioxidant capacity under stress [24,53]. Under well-watered conditions, where basal ROS levels are lower, the 10 ppm dose acted as an eustress signal that slightly promoted phenolic biosynthesis, while 20–50 ppm reduced TPC, in line with plant hormesis (low-dose stimulation, high-dose inhibition) and with evidence that elevated Se can dampen ROS-dependent signaling or even act pro-oxidatively at higher doses [54].

In comparison with total phenolics, flavonoids displayed a partially different response to SeNPs. Under drought, accumulation was stimulated only at 50 ppm, whereas lower doses (10–20 ppm) inhibited flavonoids, pointing to a selective regulation within the phenylpropanoid pathway. This suggests that SeNPs at moderate concentrations may divert precursors toward other branches (e.g., lignin or tannins), while higher doses under stress conditions restore flavonoid biosynthesis as part of an antioxidant strategy. Similar Se-driven rerouting toward the phenylpropanoid/lignin arm has been documented, for example, nano-Se stimulating phenylpropanoid metabolism and lignin precursors in red pitaya [53], while Se forms altering lignin deposition in alfalfa (*Medicago sativa* L.) [5]. Under well-watered conditions, both 10 and 20 ppm significantly promoted flavonoid accumulation, while 50 ppm reduced them to basal levels, consistent with a hormetic dose–response and aligning with reports that SeNPs upregulate flavonoid pathway genes/accumulation at low–moderate doses but may suppress secondary metabolism when the dose exceeds the optimal window [55,56].

Taken together, these assays demonstrate that SeNPs act as a redox modulator in a dose- and environment-dependent manner. Under drought stress, where ROS levels are naturally elevated, SeNPs consistently enhanced radical scavenging and reducing capacities, particularly at 10 and 50 ppm. This indicates reinforcement of both enzymatic and non-enzymatic antioxidant defenses, consistent with increased accumulation of phenolics and flavonoids at specific doses. Conversely, under well-watered conditions, where basal ROS is low, SeNPs displayed a hormetic response: 10 ppm acted as a beneficial eustress signal that maintained or slightly enhanced antioxidant activity, while higher concentrations (20–50 ppm) suppressed it, possibly due to excessive reduction in ROS signaling or even pro-oxidant effects at elevated selenium levels [24,47,57]. At the mechanistic level, integrative -omics evidence shows Se/SeNPs upregulate PAL (phenylalanine ammonia-lyase), CHS (chalcone synthase) and other nodes of phenylpropanoid/flavonoid biosynthesis, while metabolomics confirms broad increases in flavonoid/phenolic intermediates under Se treatments, providing a molecular rationale for the dose- and environment-dependent tuning observed in this study [56,58]. It should be mentioned that although SeNPs have a wider concentration range than ionic Se, excess Se can turn into a pro-oxidant effect and suppress chloroplast functions and secondary metabolism [59], which is also supported by our results that at doses of 20–50 ppm antioxidants were reduced in well-watered broccoli plants.

This biphasic dose-dependent effect reflects the classic hormetic response to Se reported in other crops under abiotic stress. Savory (*Satureja hortensis*) improves growth traits, proline levels, and antioxidant activities under heavy-metal stress, where Se improved antioxidant defenses [60], and tomato under drought, where low-dose Se enhanced photosynthetic and redox traits [61].

Unlike most previous studies, which primarily evaluated selenium-induced changes in antioxidant enzymes (e.g., SOD, CAT, POD, APX) and selected metabolites such as proline or phenolics, the present work integrates multiple chemical antioxidant assays (DPPH, FRAP, TEAC, CUPRAC). Such assays provide complementary information on the overall redox buffering capacity of plant extracts, beyond the activity of individual enzymes. For example, Se supplementation in wheat under salinity stress increased SOD and CAT activities together with phenolics and flavonoids, but antioxidant potential was inferred indirectly [48]. Similarly, studies in tomato and alfalfa under drought or heavy-metal stress reported enhanced enzymatic antioxidants after Se or SeNPs treatment, without assessing global chemical reducing capacity [42,62]. By combining classical biochemical markers with radical-scavenging and reducing assays, our study highlights the context-dependent nature of SeNPs’ antioxidant effects: low doses acted as eustress signals under well-watered conditions, whereas under drought, both 10 and 50 ppm markedly enhanced total reducing power. This integrated view underscores that SeNPs modulate antioxidant defenses at multiple levels, spanning enzyme activity, metabolite accumulation, and bulk chemical redox capacity, providing a more comprehensive picture of plant resilience to drought stress.

In addition to hormonal regulation, the antioxidant enzymes represent a frontline defense against drought-induced ROS. Among these, PODs are key antioxidant enzymes involved in scavenging hydrogen peroxide and strengthening cell walls by catalyzing lignin formation [63]. In this study, drought stress induced a significant increase in POD activity, particularly when plants were treated with SeNPs. The strong stimulation at 20 ppm SeNPs under drought suggests that selenium nanoparticles enhanced the enzymatic antioxidant defense, thereby reducing oxidative stress and contributing to the lower MDA levels observed in parallel. Such synergistic responses between enzymatic (POD) and non-enzymatic (phenolics, carotenoids) antioxidants reflect a coordinated protective mechanism. Under well-watered conditions, POD activity was much lower, and SeNPs caused only a moderate increase. This difference indicates that the stimulatory effect of SeNPs on POD is largely stress-dependent, being more pronounced under high ROS pressure. Similar results have been reported in other crops, where selenium supplementation upregulated POD activity under drought or salinity stress, but had weaker effects in non-stressed plants [42,48]. Overall, nanoselenium application enhanced POD activity in a dose-dependent manner, with the most effective induction at 20 ppm under drought stress. This enzymatic likely contributed to mitigating oxidative damage, complementing the reduction in lipid peroxidation and supporting stress tolerance.

Proline is a well-established osmoprotectant that accumulates under drought stress to stabilize proteins, protect membranes, and maintain osmotic balance [64]. In this study, drought control plants exhibited the highest proline levels, confirming its role as a marker of water deficit. Interestingly, SeNPs application under drought reduced proline accumulation at all doses, suggesting that SeNPs mitigated oxidative and osmotic stress, thereby lowering the need for excessive proline buildup. Such reductions in stress markers have been reported when Se enhances antioxidant enzyme activities and improves water status, thereby decreasing reliance on osmolyte accumulation [42,65]. At the biochemical level, the decrease in proline accumulation under SeNPs treatment probably indicates a reduction in drought-induced oxidative stress, since it has been shown to lower reactive oxygen species and lipid peroxidation, while boosting antioxidant defenses [8]. As a result, the decreased need for proline bio-synthesis as an osmoprotectant and redox buffer could account for the lower proline levels seen in SeNPs-treated plants during drought stress [39].

Under well-watered conditions, proline levels were low, as expected, but SeNPs at 10 and 20 ppm induced a moderate increase, consistent with a eustress response. This mild accumulation of proline in non-stressed plants may reflect the hormetic action of SeNPs, whereby low-to-moderate doses trigger adaptive responses, priming plants against potential stress [66]. At 50 ppm, proline returned to baseline, possibly due to suppression of ROS signaling or metabolic diversion toward other antioxidant systems.

While phenolics and flavonoids illustrate how SeNPs modulate secondary metabolism and antioxidant potential, osmolytes offer a complementary perspective on stress mitigation. Proline, in particular, is a well-established osmoprotectant and marker of drought stress, making it a valuable parameter for understanding how SeNPs influence both osmotic balance and chlorophyll metabolism. Proline is not a direct precursor of chlorophyll, but its metabolism is tightly interconnected with that of tetrapyrroles through glutamate. Glutamate represents the common starting point for both proline biosynthesis (via P5CS and P5CR) and chlorophyll biosynthesis (via GluRS, GluTR, and GSAT, leading to the formation of δ-aminolevulinic acid, the universal precursor of porphyrins) [67,68]. Under abiotic stress conditions, proline accumulation acts as an adaptive mechanism but may decrease the availability of glutamate for the tetrapyrrole pathway, contributing to a reduction in chlorophyll content. On the other hand, its osmoprotective and antioxidant functions limit pigment degradation and protect the chloroplast against ROS. Therefore, proline exerts a dual impact on photosynthesis: metabolically, through competition for glutamate, and protectively, by maintaining the structural and functional integrity of the photosynthetic apparatus [69].

Overall, these findings highlight that SeNPs modulate proline metabolism in a dose- and context-dependent manner: it reduces proline under drought by alleviating stress severity, while under well-watered conditions it acts as a mild elicitor at low doses but loses effectiveness at higher concentrations.

In addition to osmolytes such as proline, oxidative stress markers provide direct evidence of membrane integrity under drought. Among these, MDA is widely used as an indicator of lipid peroxidation and oxidative damage.

The accumulation of MDA under drought stress in control plants reflects enhanced membrane lipid peroxidation caused by oxidative damage, a well-known consequence of water deficit [70]. The significant reduction in MDA in SeNPs-treated plants indicates a protective role of SeNPs against oxidative stress, likely by boosting the antioxidant defense system and stabilizing cell membranes. This effect was dose-dependent, with the strongest reduction observed at 20–50 ppm, suggesting that SeNPs at moderate-to-high concentrations effectively suppressed lipid peroxidation.

In terms of mechanism, the decrease in MDA can be explained by a reinforced antioxidant network that interrupts lipid peroxidation chains. Carotenoids preserved under SeNPs can quench singlet oxygen and triplet chlorophyll, prevent the initiation of lipid peroxidation and protect membrane integrity [71]. Simultaneously, phenolic compounds, boosted by SeNPs, act as potent radical scavengers, especially of peroxyl radicals (ROO•), effectively terminating chain reactions of lipid oxidation [72]. Additionally, enhanced activities of enzymatic antioxidants such as SOD, CAT, GPX reduce ROS accumulation, thereby lowering the incidence of oxidative initiation events. Together, these components stabilize membranes structurally and functionally, which aligns with the significantly lower MDA levels observed in SeNPs-treated broccoli.

Under well-watered conditions, basal levels of MDA were lower, and SeNPs application further reduce lipid peroxidation. This suggests that, beyond its role under stress, SeNPs may contribute to maintaining redox homeostasis even in non-stressed plants, although the magnitude of the effect was smaller than under drought.

Overall, these results demonstrate that SeNPs mitigate drought-induced oxidative membrane damage by reducing MDA accumulation, consistent with its role as a redox modulator enhancing both enzymatic and non-enzymatic antioxidant defenses. Similar protective effects of selenium against lipid peroxidation have been reported in several crops under abiotic stresses, including reductions in MDA and H_2_O_2_ in canola under drought [27] and decreased MDA in grapevine saplings treated with 10 ppm Se-NP [24], as well as in wheat and tomato [42,48].

Beyond metabolites and oxidative markers, plant hormones provide a regulatory layer that integrates growth and stress responses. Among them, IAA is particularly relevant. IAA is the main auxin regulating plant growth and development, including cell elongation, differentiation, and root architecture [73]. Its role under drought is less direct than that of abscisic acid, but auxin levels influence stress adaptation through modulation of root growth and interaction with antioxidant and osmolyte responses [74]. In this study, drought stress reduced IAA compared to well-watered conditions, consistent with previous reports that water deficit suppresses auxin biosynthesis and transport [75]. Interestingly, SeNPs application reversed this trend under drought, significantly increasing IAA levels at 10–20 ppm. This observation is aligned with reports indicating that selenium nanoparticles can modulate auxin metabolism. In *Gerbera jamesonii*, Khai et al. [26] demonstrated that SeNPs treatments enhanced levels of endogenous hormones, including IAA, cytokinins, and gibberellins, coinciding with improved rooting and antioxidant activity under acclimatization conditions [26]. Similary, SeNPs combined with melatonin markedly increased IAA accumulation in melon seedlings (+236–291%) [25]. These findings support the hypothesis that SeNPs may stimulate or stabilize IAA biosynthesis, thereby enhancing root development and contributing to stress adaptation under drought. However, direct effects on root morphology were not assessed in the present study. In contrast, under well-watered conditions, IAA was highest in the untreated control and decreased under SeNPs treatments, especially at 10 ppm. This could reflect a hormetic response, where in the absence of stress, SeNPs downregulates auxin metabolism to avoid unnecessary stimulation of growth pathways. The partial recovery at 20–50 ppm may indicate dose-dependent modulation, but overall, the effect of SeNPs under well-watered conditions was inhibitory for IAA. Thus, SeNPs appear to exert a context-dependent regulation of auxin metabolism, stimulating IAA under drought but reducing it under non-stressed conditions.

Leaf mass per area is a widely used indicator of leaf structural investment, integrating lamina thickness and tissue density, and is known to increase under drought as part of an adaptive adjustment to reduced water availability [76,77]. In our study, drought-induced thickening was evident in control plants; however, the application of NSe altered this structural response. Notably, 50 ppm NSe under drought resulted in the lowest LMA, suggesting a possible attenuation of leaf densification. This pattern implies that nanoselenium may influence leaf structure through mechanisms that are at least partly independent of the biochemical stress pathways, potentially modulating growth or cell-wall-associated processes in stressed tissues.

Overall, the combined analysis of relative responses across pigments, phenolics, antioxidant capacity, stress markers, and IAA highlight an apparent hormetic and context-dependent effect of SeNPs. Under drought stress, SeNPs treatments at 10–50 ppm generally reduced oxidative damage (lower MDA and proline) while enhancing protective pigments (chlorophyll a and carotenoids), phenolic compounds, radical scavenging capacity, and peroxidase activity. This integrated response points to a coordinated adjustment of both enzymatic and non-enzymatic antioxidant defenses, resulting in reduced stress severity. Under well-watered conditions, however, the relative values reveal an opposite trend: only low doses (10 ppm) act as mild eustress, stimulating phenolics, flavonoids, and antioxidant activity, whereas higher doses suppress these pathways and even reduce IAA. These findings demonstrate that SeNPs acts as a dual regulator, alleviating drought-induced oxidative stress while displaying hormetic behavior under optimal growth, with the “optimum dose” shifting according to water regime. The relative-response approach thus provides a clearer view of the dose–environment interactions, underscoring the potential of SeNPs as a fine-tuning agent for plant stress physiology.

## 5. Conclusions

This study provides comprehensive evidence that SeNPs mitigate drought-induced oxidative damage in broccoli by coordinating the modulation of photosynthetic pigments, secondary metabolites, antioxidant capacity, osmolyte accumulation, lipid peroxidation, and hormonal balance. Under drought stress, SeNPs enhanced chlorophyll a content, phenolics, flavonoids, and antioxidant capacity, while reducing MDA and proline levels, thereby supporting photosynthetic integrity and membrane stability. At the enzymatic level, SeNPs stimulated POD activity, suggesting enhanced ROS detoxification capacity. IAA levels were positively regulated by SeNPs under drought stress, suggesting a role in supporting growth-related physiological processes and stress adaptation.

Under well-watered conditions, SeNPs exhibited a hormetic response, with low doses (10 ppm) acting as beneficial eustress by promoting antioxidant-related metabolites, whereas higher concentrations suppressed these responses and reduced IAA. This highlights the context-dependent nature of SeNPs action, with the optimal dose shifting according to water availability.

Overall, SeNPs emerge as an effective redox-modulating agent rather than strategy for enhancing drought tolerance in broccoli by fine-tuning both enzymatic and non-enzymatic antioxidant defenses. The contrasting responses observed under drought versus well-watered conditions emphasize the importance of dose optimization to maximize physiological benefits while avoiding inhibitory effects.

## Figures and Tables

**Table 1:** Description of the treatments used in the study.

Description	Label
Control under well-watered conditions without SeNPs treatment	WW_0
SeNPs treatment at 10 ppm under well-watered conditions	WW_10
SeNPs treatment at 20 ppm under well-watered conditions	WW_20
SeNPs treatment at 50 ppm under well-watered conditions	WW_50
Control under drought stress without SeNPs treatment	DS_0
SeNPs treatment at 10 ppm under drought stress	DS_10
SeNPs treatment at 20 ppm under drought stress	DS_20
SeNPs treatment at 50 ppm under drought stress	DS_50

**Table 2:** Morphophysiological parameters (Height, Stem diameter, and SPAD index) of broccoli plants measured at five time points T1 (initial), T2 (7 days), T3 (14 days), T4 (21 days), and T5 (28 days) under well-watered (WW) and drought stress (DS) conditions following weekly foliar applications of selenium nanoparticles (SeNPs) at 10, 20, and 50 ppm. Values are presented as mean ± SD (*n* = 5).

		Treatment							
		DS_0	DS_10	DS_20	DS_50	WW_0	WW_10	WW_20	WW_50
Height (cm)	T1	87.53 ± 1.43 ^a^	86.25 ± 9.67 ^a^	84.80 ± 4.40 ^a^	87.53 ± 1.22 ^a^	84.88 ± 7.85 ^a^	84.40 ± 5.08 ^a^	85.34 ± 2.91 ^a^	88.50 ± 5.17 ^a^
	T2	92.12 ± 5.33 ^a^	92.16 ± 10.08 ^a^	86.10 ± 3.78 ^a^	91.90 ± 9.12 ^a^	98.04 ± 10.71 ^a^	86.78 ± 2.59 ^a^	87.82 ± 3.96 ^a^	91.78 ± 3.99 ^a^
	T3	95.38 ± 6.83 ^ab^	101.58 ± 13.07 ^ab^	86.80 ± 5.07 ^b^	96.40 ± 14.93 ^ab^	111.78 ± 6.97 ^a^	105.64 ± 4.47 ^ab^	98.16 ± 6.52 ^ab^	103.26 ± 17.26 ^ab^
	T4	100.10 ± 17.37 ^bc^	105.40 ± 14.38 ^bc^	92.20 ± 6.61 ^c^	103.94 ± 16.24 ^bc^	131.32 ± 9.13 ^a^	114.34 ± 2.13 ^ab^	102.46 ± 7.87 ^bc^	111.06 ± 18.32 ^bc^
	T5	103.67 ± 19.13 ^bc^	110.60 ± 15.53 ^bc^	102.80 ± 9.83 ^c^	112.40 ± 17.92 ^bc^	135.20 ± 13.18 ^a^	123.20 ± 2.28 ^ab^	107.50 ± 11.15 ^bc^	121.60 ± 19.88 ^abc^
Stem (mm)	T1	7.27 ± 0.51 ^a^	7.49 ± 1.00 ^a^	7.27 ± 0.45 ^a^	7.23 ± 0.63 ^a^	7.48 ± 0.70 ^a^	7.32 ± 0.74 ^a^	7.23 ± 0.13 ^a^	7.40 ± 0.56 ^a^
	T2	7.66 ± 0.6 ^a^	7.70 ± 0.74 ^a^	7.77 ± 0.32 ^a^	7.53 ± 0.48 ^a^	7.96 ± 0.83 ^a^	7.57 ± 0.82 ^a^	7.41 ± 0.19 ^a^	7.98 ± 0.66 ^a^
	T3	7.55 ± 1.02 ^a^	7.77 ± 0.75 ^a^	7.84 ± 0.33 ^a^	7.70 ± 0.45 ^a^	8.05 ± 0.85 ^a^	7.64 ± 0.81 ^a^	7.58 ± 0.18 ^a^	8.09 ± 0.62 ^a^
	T4	9.20 ± 0.49 ^ab^	8.76 ± 0.91 ^ab^	8.67 ± 0.24 ^ab^	8.35 ± 0.34 ^b^	9.75 ± 0.85 ^a^	9.08 ± 1.05 ^ab^	8.36 ± 0.44 ^b^	8.61 ± 0.56 ^ab^
	T5	9.61 ± 1.08 ^abc^	8.97 ± 0.71 ^bc^	9.21 ± 0.42 ^c^	8.78 ± 0.27 ^c^	10.74 ± 0.76 ^a^	10.40 ± 0.64 ^ab^	9.08 ± 1.05 ^c^	8.84 ± 0.55 ^c^
SPAD index	T1	51.38 ± 7.22 ^a^	50.06 ± 5.76 ^a^	46.16 ± 2.87 ^a^	48.02 ± 5.84 ^a^	47.68 ± 5.21 ^a^	50.62 ± 2.70 ^a^	46.12 ± 1.46 ^a^	46.92 ± 3.99 ^a^
	T2	49.74 ± 2.69 ^a^	48.28 ± 5.21 ^a^	46.02 ± 3.23 ^a^	48.72 ± 2.06 ^a^	49.98 ± 1.72 ^a^	49.04 ± 2.11 ^a^	48.20 ± 2.28 ^a^	45.82 ± 3.04 ^a^
	T3	56.84 ± 6.54 ^a^	54.66 ± 2.32 ^a^	54.76 ± 3.73 ^a^	55.98 ± 1.00 ^a^	56.00 ± 3.78 ^a^	55.68 ± 3.01 ^a^	52.22 ± 4.50 ^a^	52.56 ± 3.03 ^a^
	T4	65.84 ± 7.48 ^ab^	57.02 ± 2.29 ^c^	58.40 ± 4.42 ^bc^	58.14 ± 1.23 ^bc^	68.08 ± 9.10 ^a^	57.66 ± 3.30 ^bc^	57.96 ± 4.08 ^bc^	55.54 ± 2.44 ^c^
	T5	70.00 ± 8.09 ^ab^	64.66 ± 4.03 ^bc^	63.54 ± 4.93 ^bc^	63.14 ± 1.87 ^bc^	73.34 ± 8.07 ^a^	71.40 ± 1.32 ^ab^	67.48 ± 2.99 ^abc^	61.42 ± 5.37 ^c^

Different letters within a column indicate significant differences at *p* < 0.05 according to Tukey’s test.

**Table 3:** Effect of drought and SeNPs treatments on photosynthetic pigments.

Treatments	Chl a (µg/mL)	Chl b (µg/mL)	Total Chl (µg/mL)	Chl a/b	CARs (µg/mL)	Total Chl/CARs
DS_0	8.18 ± 0.02 ^a^	5.13 ± 0.01 ^a^	13.31 ± 0.04 ^a^	1.59 ± 0.00 ^a^	1.26 ± 0.02 ^a^	10.54 ± 0.16 ^b^
DS_10	11.09 ± 0.02 ^d^	6.31 ± 0.02 ^c^	17.40 ± 0.03 ^d^	1.76 ± 0.00 ^b^	2.18 ± 0.01 ^e^	7.97 ± 0.04 ^e^
DS_20	10.76 ± 0.01 ^c^	6.24 ± 0.02 ^c^	17.00 ± 0.03 ^c^	1.73 ± 0.00 ^b^	2.00 ± 0.01 ^d^	8.50 ± 0.04 ^d^
DS_50	10.41 ± 0.01 ^b^	5.96 ± 0.02 ^b^	16.37 ± 0.03 ^b^	1.75 ± 0.00 ^b^	1.91 ± 0.02 ^c^	8.58 ± 0.06 ^d^
WW_0	11.80 ± 0.02 ^e^	7.18 ± 0.02 ^e^	18.98 ± 0.02 ^f^	1.65 ± 0.01 ^a^	1.98 ± 0.03 ^d^	9.58 ± 0.11 ^c^
WW_10	12.01 ± 0.28 ^e^	7.63 ± 0.18 ^f^	19.64 ± 0.09 ^g^	1.58 ± 0.07 ^a^	2.14 ± 0.06 ^e^	9.18 ± 0.23 ^c^
WW_20	12.01 ± 0.28 ^e^	7.61 ± 0.17 ^f^	19.55 ± 0.19 ^g^	1.57 ± 0.08 ^a^	1.84 ± 0.06 ^c^	10.63 ± 0.24 ^b^
WW_50	11.15 ± 0.04 ^d^	6.95 ± 0.03 ^d^	18.11 ± 0.02 ^e^	1.60 ± 0.01 ^a^	1.63 ± 0.04 ^b^	11.09 ± 0.28 ^a^
Mean	10.93 ± 0.19	6.63 ± 0.13	17.55 ± 0.31	1.65 ± 0.01	1.86 ± 0.05	9.51 ± 1.10
MS (Treatment)	7.859 **	3.800 **	21.715 **	0.031 **	0.448 **	4.091 **
MS (Error)	0.020	0.008	0.006	0.001	0.001	0.033

Note: Values are means ± SD (*n* = 3). Different letters within a column indicate significant differences at *p* < 0.05 according to Tukey’s test. Levels of significance: ** *p* < 0.01, ns = not significant. Chl a: Chlorophyll a, Chl b: Chlorophyll b, Total Chl: Total chlorophyll, Chl a/b: Chlorophyll a to b ratio, CARs: Carotenoids.

**Table 4:** Effect of drought and SeNPs treatments on polyphenols, flavonoids and antioxidant activity.

Treatments	TPC (mg GAE/g FW)	TFC (mg QE/g FW)	DPPH (µmol TE/g FW)	FRAP (µmol TE/g FW)	TEAC (µmol TE/g FW)	CUPRAC (µmol TE/g FW)
DS_0	3.41 ± 0.20 ^bc^	7.26 ± 0.77 ^d^	5.60 ± 0.60 ^b^	18.47 ± 0.35 ^a^	11.64 ± 1.41 ^a^	221.30 ± 5.38 ^a^
DS_10	4.08 ± 0.24 ^c^	5.59 ± 0.27 ^c^	19.75 ± 0.26 ^ef^	29.16 ± 3.63 ^c^	19.48 ± 1.54 ^b^	266.30 ± 36.25 ^ef^
DS_20	3.57 ± 0.04 ^bc^	4.19 ± 0.26 ^b^	10.54 ± 0.59 ^c^	20.49 ± 0.56 ^ab^	17.55 ± 0.38 ^b^	244.43 ± 1.50 ^bc^
DS_50	6.01 ± 0.67 ^d^	7.75 ± 0.75 ^d^	22.14 ± 0.26 ^f^	24.13 ± 1.12 ^bc^	18.96 ± 0.25 ^b^	252.43 ± 6.50 ^cd^
WW_0	3.15 ± 0.64 ^bc^	0.78 ± 0.61 ^a^	15.23 ± 2.22 ^d^	20.22 ± 0.56 ^ab^	18.06 ± 1.15 ^b^	260.18 ± 0.50^de^
WW_10	3.43 ± 0.60 ^bc^	3.73 ± 0.21 ^b^	18.81 ± 0.17 ^e^	20.63 ± 2.10 ^ab^	18.06 ± 0.38 ^b^	273.73 ± 3.02 ^f^
WW_20	1.43 ± 0.16 ^a^	5.77 ± 0.13 ^c^	2.70 ± 0.09 ^a^	18.12 ± 0.28 ^a^	12.66 ± 0.38 ^a^	245.68 ± 0.75 ^bc^
WW_50	2.41 ± 0.64 ^ab^	1.47 ± 0.43 ^a^	7.39 ± 1.02 ^b^	20.08 ± 3.49 ^ab^	12.54 ± 0.51 ^a^	239.93 ± 1.25 ^b^
Mean	3.44 ± 1.32	4.57 ± 2.45	12.77 ± 6.95	21.41 ± 3.83	16.12 ± 3.19	250.49 ± 16.06
MS (Treatment)	5.228 **	19.137 **	156.873 **	39.219 **	31.643 **	819.170 **
MS (Error)	0.218	0.238	0.855	3.982	0.812	12.255

Note: Values are means ± SD (*n* = 3). Different letters within a column indicate significant differences at *p* < 0.05 according to Tukey’s test. Levels of significance: ** *p* < 0.01. TPC, total phenolic content; TFC, total flavonoid content; DPPH, radical scavenging capacity determined by the DPPH assay; FRAP, ferric reducing antioxidant power; TEAC, Trolox equivalent antioxidant capacity; CUPRAC, cupric reducing antioxidant capacity.

**Table 5:** Effects of foliar-applied green-synthesized nanoselenium particles (SeNPs) on oxidative stress (MDA), osmolyte (Proline), enzymatic antioxidant (POD), and hormonal parameters (IAA) in broccoli (*Brassica oleracea* var. *italica*) grown under well-watered (WW) and drought-stressed (DS) conditions.

Treatments	MDA (µmol/g FW)	Proline (µmol/g FW)	POD (U/g FW)	IAA (nmol/g FW)
DS_0	26.22 ± 3.36 ^e^	3.83 ± 0.07 ^e^	52.46 ± 1.15 ^b^	145.00 ± 4.59 ^a^
DS_10	19.20 ± 0.95 ^cd^	2.75 ± 0.03 ^c^	71.38 ± 0.29 ^f^	203.30 ± 8.51 ^d^
DS_20	15.38 ± 0.31 ^abc^	3.23 ± 0.03 ^d^	102.48 ± 1.58 ^g^	212.80 ± 6.22 ^de^
DS_50	15.22 ± 0.45 ^ab^	3.03 ± 0.09 ^cd^	68.22 ± 0.86 ^ef^	164.70 ± 3.28 ^ab^
WW_0	19.56 ± 0.84 ^d^	1.32 ± 0.21 ^a^	40.13 ± 0.29 ^a^	229.90 ± 4.91 ^e^
WW_10	18.99 ± 0.39 ^bcd^	2.84 ± 0.08 ^b^	59.34 ± 0.00 ^c^	172.90 ± 0.33 ^bc^
WW_20	14.75 ± 1.16 ^a^	2.34 ± 0.18 ^b^	66.50 ± 2.29 ^de^	196.10 ± 1.97 ^cd^
WW_50	15.24 ± 0.54 ^ab^	1.23 ± 0.21 ^a^	63.21 ± 1.00 ^d^	194.80 ± 22.27 ^cd^
Mean	18.07 ± 0.79	2.50 ± 0.18	65.47 ± 3.51	189.96 ± 5.59
MS (Treatment)	45.082 **	2.441 **	971.692 **	2273.934 **
MS (Error)	1.873	0.018	1.371	83.405

Note: Values are means ± SD (*n* = 3). Different letters within a column indicate significant differences at *p* < 0.05 according to Tukey’s test. Levels of significance: **: *p* < 0.01. MDA: Malondialdehyde, Proline: Proline, POD: Peroxidase activity, IAA: Indole-3-acetic acid, DS: drought stress; WW: well-watered. IAA was evaluated as a growth-related hormonal indicator rather than a drought stress marker.

## Data Availability

All relevant data are presented in the article. Additional raw data are available from the corresponding authors upon reasonable request.

## Supplementary Materials

The following supporting information can be downloaded at: https://www.mdpi.com/article/10.3390/life16010070/s1, Figure S1: UV–Vis absorption spectrum of green-synthesized selenium nanoparticles (SeNPs), showing a characteristic absorption maximum at approximately 270 nm.

## Abbreviations

The following abbreviations are used in this manuscript:

Chl	Chlorophyll
CUPRAC	Cupric reducing antioxidant capacity
DLS	Dynamic light scattering
DPPH	2,2-diphenyl-1-picrylhydrazyl
DS	Drought-stressed conditions
EDAX	Energy Dispersive X-ray Spectroscopy
FC	Field capacity
FRAP	Ferric Reducing Antioxidant Power
FW	Fresh weight
IAA	Indole-3-acetic acid
MDA	Malondialdehyde
SeNPs	Selenium nanoparticles
POD	Peroxidase
TEAC	Trolox Equivalent Antioxidant Capacity
TEM	Transmission electron microscopy
TFC	Total flavonoid content
TPC	Total phenolic content
TPTZ	2,4,6-tripyridyl-s-triazine
WW	Well-watered
