# Supplementary material for: Foliar Application of Nanoselenium Enhances Drought Tolerance in Brassica oleracea var. italica Through Antioxidant Reinforcement and Pigment Stabilization
Source: Life (Basel). 2026 Jan 2;16(1):70. doi: 10.3390/life16010070 (PMC12843310; doi:10.3390/life16010070)
Supplement: Supplementary file 1 [file life-16-00070-s001.zip › life-4053437-supplementary.pdf]

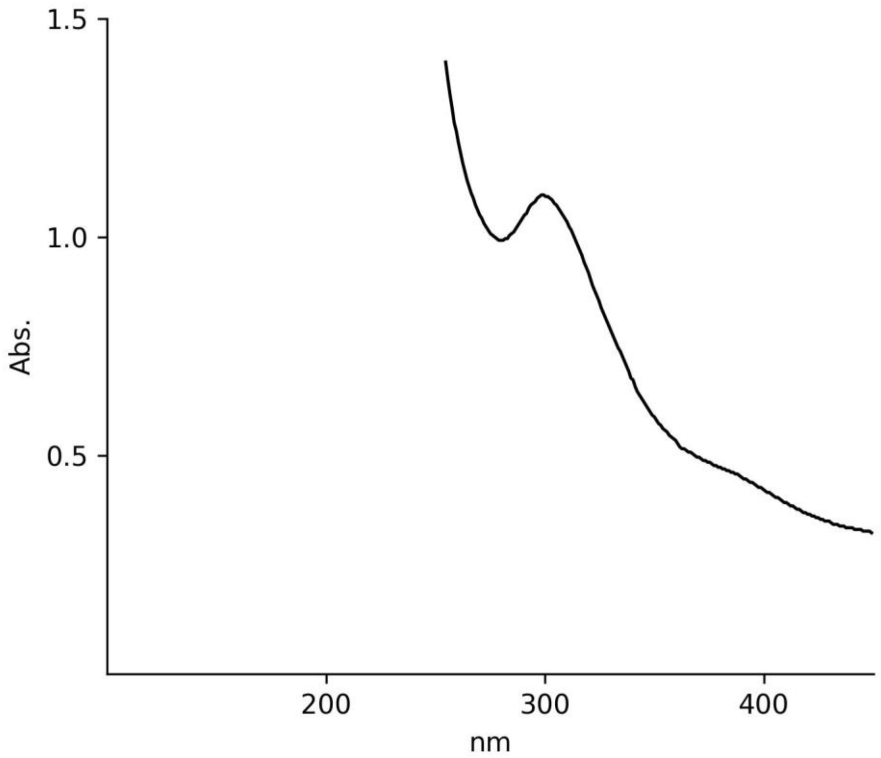

Figure S1. UV–Vis absorption spectrum of green-synthesized selenium nanoparticles (SeNPs), showing a characteristic absorption maximum at approximately 270 nm.
